# Efficacy and safety of leuprorelin acetate 6-month depot, TAP-144-SR (6M), in combination with tamoxifen in postoperative, premenopausal patients with hormone receptor-positive breast cancer: a phase III, randomized, open-label, parallel-group comparative study

**DOI:** 10.1007/s12282-016-0691-6

**Published:** 2016-03-26

**Authors:** Junichi Kurebayashi, Tatsuya Toyama, Shuuji Sumino, Eri Miyajima, Tsukasa Fujimoto

**Affiliations:** 1 Department of Breast and Thyroid Surgery, Kawasaki Medical School, 577 Matsushima, Kurashiki, Okayama 701-0192 Japan; 2 Department of Breast and Endocrine Surgery, Nagoya City University Hospital, Nagoya, Aichi Japan; 3 Takeda Pharmaceutical Company Limited, Osaka, Japan

**Keywords:** Leuprorelin acetate 6-month depot, Luteinizing hormone–releasing hormone agonist, Premenopausal hormone receptor-positive breast cancer, Adjuvant endocrine therapy, Ovarian function suppression

## Abstract

**Background:**

Leuprorelin acetate, a luteinizing hormone-releasing hormone agonist, is used worldwide in premenopausal women with hormone receptor-positive breast cancer. This study was conducted to assess the non-inferiority of the 6-month depot formulation, TAP-144-SR (6M) 22.5 mg to the 3-month depot formulation, TAP-144-SR (3M) 11.25 mg in postoperative, premenopausal patients with hormone receptor-positive breast cancer.

**Methods:**

This was a 96-week phase III, randomized, open-label, parallel-group comparative study. All patients concomitantly received oral tamoxifen (20 mg daily). The primary endpoint was the suppression rate of serum estradiol (E_2_) to the menopausal level (≤30 pg/mL) from Week 4 through Week 48.

**Results:**

In total, 167 patients were randomized to receive TAP-144-SR (6M) (*n* = 83) or TAP-144-SR (3M) (*n* = 84) and the E_2_ suppression rate was 97.6 and 96.4 %, respectively. The estimated between-group difference was 1.2 % (95 % confidence interval −5.2 to 7.8). The non-inferiority of TAP-144-SR (6M) to TAP-144-SR (3M) for E_2_ suppression was confirmed. As for safety, common adverse events were hot flush and injection site reactions including induration, pain, and erythema in both treatment groups, which were of ≤Grade 2 in severity and not serious. No significant between-group differences in safety profiles and tolerability were observed.

**Conclusions:**

TAP-144-SR (6M) was not inferior to TAP-144-SR (3M) for its suppressive effect on serum E_2_. TAP-144-SR (6M) was also as well tolerated as TAP-144-SR (3M).

## Introduction

For the treatment of premenopausal women with hormone receptor-positive breast cancer, it is highly important to suppress estrogen production through ovarian function suppression (OFS). OFS therapy with a luteinizing hormone–releasing hormone (LH–RH) agonist, in combination with adjuvant tamoxifen or chemotherapy is widely used for postoperative premenopausal endocrine-responsive breast cancer patients [1–7]. However, the optimal treatment duration of postoperative adjuvant endocrine therapy with an LH–RH agonist alone or in combination with tamoxifen is still controversial [8–10]. Although premenopausal hormone receptor-positive breast cancer patients have a relatively good prognosis, the risk of recurrence remains at 5 years or longer after surgery, suggesting the importance of long-term endocrine therapy for 5 years or longer for the treatment of patients at high risk for recurrence [11].

Leuprorelin acetate (leuprorelin), an LH–RH agonist is commonly used for the treatment of patients with hormone-responsive prostate cancer and premenopausal breast cancer worldwide. It is available in 1- and 3-month depot formulations for both cancers, and a 6-month depot formulation for prostate cancer.

A 6-month depot formulation, TAP-144-SR (6M), was initially developed for prostate cancer in Japan and a phase II study was conducted in treatment-naïve prostate cancer patients. The results showed that the optimal clinical dosage of TAP-144-SR (6M) in Japan is 22.5 mg [12].

In parallel with the phase III study in prostate cancer, this phase III study was also conducted in Japan for the first time to assess the non-inferiority of TAP-144-SR (6M) 22.5 mg to TAP-144-SR (3M) 11.25 mg regarding its suppressive effect on serum estradiol (E_2_), and to evaluate its efficacy and safety in postoperative, premenopausal patients with hormone receptor-positive breast cancer.

## Patients and methods

### Study design

A phase III, randomized, open-label, parallel-group comparative study of TAP-144-SR (6M) to TAP-144-SR (3M) was conducted to evaluate the efficacy, safety, and pharmacokinetics of the 2 formulations and hormone levels in postoperative, premenopausal patients with endocrine-responsive breast cancer. Following a 4-week screening period, eligible patients were randomly assigned at a 1:1 ratio to receive injection of either TAP-144-SR (6M) 22.5 mg (6M group) or TAP-144-SR (3M) 11.25 mg (3M group) for 96 weeks using dynamic allocation with the number of positive axillary lymph nodes (0, 1–3, ≥4), tumor diameter (≤2.0, >2.0 cm), estrogen receptor (ER)/progesterone receptor (PgR) status (ER+/PgR+, ER+/PgR−, ER−/PgR+), age (at the time of consent; ≤39, 40–44, ≥45 years), pre- and post-operative chemotherapy (presence, absence), and study site as factors. All patients in both groups concomitantly received oral tamoxifen citrate (20 mg daily) throughout the 96-week study period.

This study was conducted in accordance with the International Conference on Harmonisation of Good Clinical Practice Guidelines, the principles of the Declaration of Helsinki, and all applicable laws and regulations, at 20 medical centers in Japan between April 2012 and December 2014. The protocol was reviewed and approved by the Institutional Review Boards of all study participating sites. All patients provided written informed consent before enrollment. The clinical trial registration number is NCT01546649.

### Patients

Japanese premenopausal patients with histologically confirmed primary breast cancer who met the following criteria were eligible: age ≥20 years; both or either ER+ or PgR+, and human epidermal growth factor receptor type 2 (HER-2)-negative primary tumor; T1–T3, any N, and M0 according to the TNM classification; any type of surgical procedure (in case of breast-conserving surgery, postoperative radiation to the breast was required); any type of preoperative and/or postoperative adjuvant chemotherapy prior to enrollment; history of regular menstruation or follicle-stimulating hormone (FSH) of <40 mIU/mL and E_2_ of ≥10 pg/mL within 12 weeks prior to enrollment and not having chemical menopause (FSH of ≥40 mIU/mL and E_2_ of <10 pg/mL) within 12 weeks after completion of the postoperative chemotherapy; capable of receiving the study drug and tamoxifen within 12 weeks after surgery or after postoperative chemotherapy completion prior to enrollment; Eastern Cooperative Oncology Group performance status of Grade 0 or 1.

Exclusion criteria included the following: endocrine therapy prior to surgery or postoperative endocrine therapy before enrollment; bilateral oophorectomy or ovarian irradiation; inflammatory breast cancer or bilateral breast cancer; non-invasive ductal carcinoma, multiple cancers or a history of cancer in other organs; QTcF interval exceeding 460 ms on the 12-lead electrocardiogram (ECG) at screening.

### Primary and secondary endpoints

The primary endpoint was the suppression rate of serum E_2_ to the menopausal level (≤30 pg/mL), which is the best index of the medicinal effect of leuprorelin, from Week 4 through Week 48. The secondary endpoints included: serum E_2_, LH, and FSH concentrations; disease-free survival [DFS; defined as the time from random assignment to disease event (recurrence, second primary cancer, or death)] and distant DFS [DDFS; defined as the time from random assignment to disease event (distant recurrence, second primary cancer, or death)] throughout the study period as measures of the long-term efficacy. All the serum hormone concentrations were measured at a central laboratory (SRL Medisearch Inc., Tokyo, Japan). Electrochemiluminescence immunoassay for E_2_ (ECLusys^®^E2III, Roche Diagnostics K.K., Tokyo, Japan), chemiluminescence immunoassay for LH (ARCHITECT^®^ · LH, Abbott Japan, Chiba, Japan) and chemiluminescence immunoassay for FSH (ARCHITECT^®^ · FSH, Abbott Japan, Chiba, Japan) were used to measure each serum hormone concentration.

Pharmacokinetic analysis was performed by measuring the serum concentrations of unchanged TAP-144 using LC/MS/MS from the start of the study drug administration through Week 48.

Safety data were obtained from the findings of clinical signs/symptoms, body weight, vital signs, laboratory test results, 12-lead ECG, and bone mineral density (BMD) measured by dual-energy X-ray absorptiometry throughout the study period. Adverse events (AEs) were recorded and graded according to the National Cancer Institute Common Terminology Criteria for Adverse Events version 4.0.

### Statistical analysis

The required sample size was estimated as 74 subjects in each treatment group, a total of 148 subjects, based on which the conditions were set as a non-inferiority margin of 10 %, with a two-sided alpha level of 0.05, ensuring 80 % power. Taking a possible drop-out rate of 10 % into consideration, 82 patients in each treatment group, thus a total of 164 patients were required.

The Full Analysis Set (FAS) was defined as all patients who were randomized and received at least 1 dose of the study drug, and the Hormone Analysis Set (HAS) was defined as the patients who had no major protocol deviations, and in whom the primary endpoints were evaluable. The FAS was used for the primary and secondary endpoints and the HAS was used to examine the robustness of the results of the primary analysis. The treatment difference [TAP-144-SR (6M) − TAP-144-SR (3M)] and the two-sided 95 % confidence interval (CI) were calculated by a method based on the Wilson score method [13]. If the lower bound of the two-sided 95 % CI was greater than the prespecified non-inferiority margin of −10 %, clinical non-inferiority of TAP-144-SR (6M) to TAP-144-SR (3M) would be declared. Summary statistics were obtained for the secondary efficacy endpoints. Serum E_2_ concentrations lower than or equal to the limit of quantification (10 pg/mL) were considered to be 0 pg/mL. DFS and DDFS were estimated by the Kaplan–Meier method.

For the safety analysis, AEs and their severity were analyzed by treatment group. AEs were summarized in the Safety Data Analysis Set (SAS) defined as all patients who were randomized and received at least 1 dose of the study drug and were coded by the System Organ Class Preferred Terms based on the Medical Dictionary for Regulatory Activities (MedDRA) terminology, version 16.1.

As for pharmacokinetic analysis, summary statistics were calculated for serum unchanged-TAP-144 concentrations through Week 48 in the Pharmacokinetics Analysis Set (PAS; defined as the population of patients in the FAS in whom serum unchanged-TAP-144 concentrations were appropriately measured), and for pharmacokinetic parameters in patients in whom serum unchanged-TAP-144 concentrations were measured at 3 and 6 h after the study drug administration in the PAS.

## Results

### Patient demographics

**Fig. 1:**
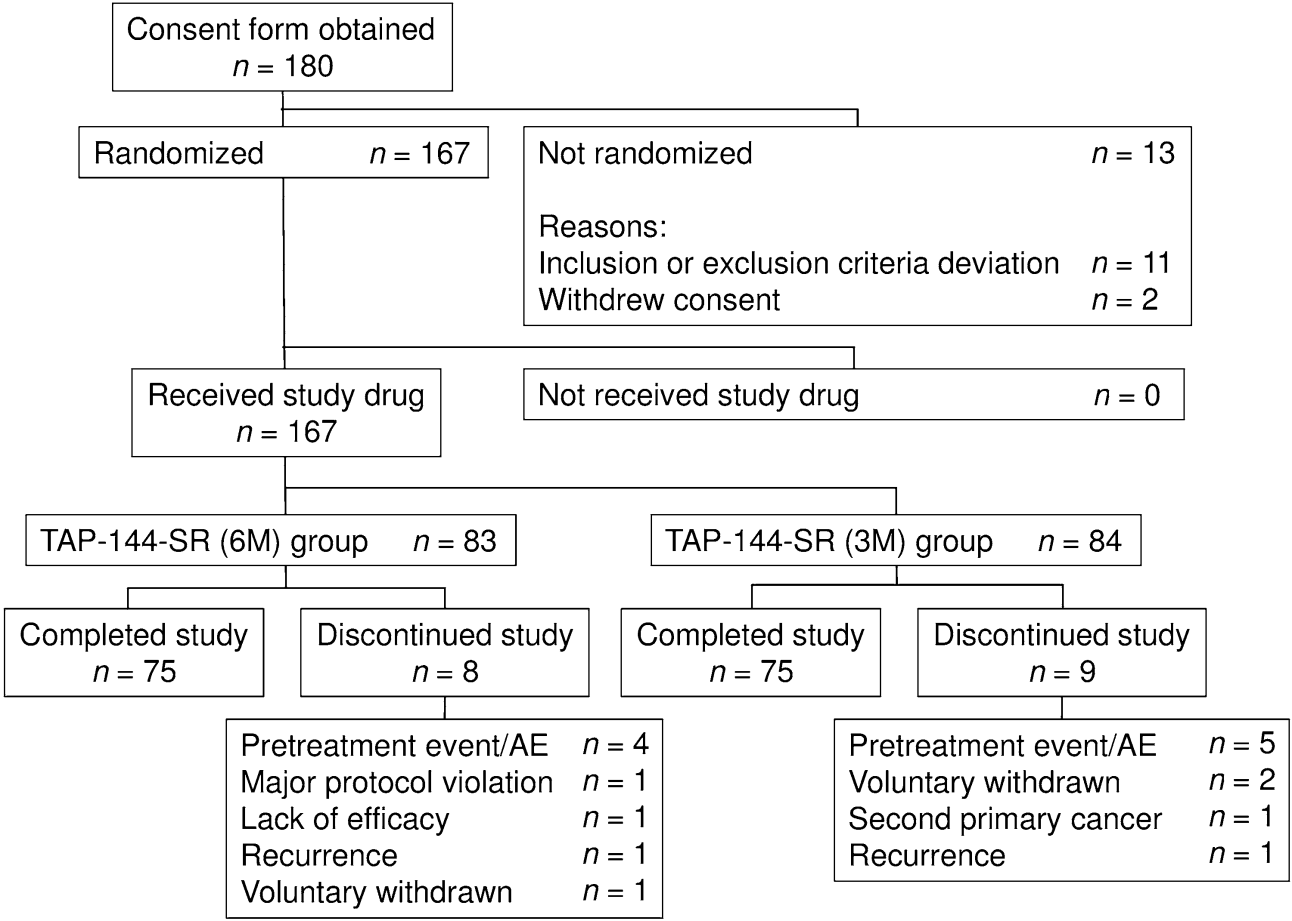
Patient disposition. *n* number of patients evaluated, *AE* adverse event

Figure 1 shows the patient disposition. Of the 180 patients who provided written informed consent, a total of 167 patients were randomized, 83 patients received TAP-144-SR (6M) and 84 patients received TAP-144-SR (3M). Overall, 150 patients (75 patients in each treatment group) completed the 96-week study treatment, and the majority of patients (92.8 and 91.7 % in the 6M and 3M groups, respectively) received the maximum doses (4 doses and 8 doses in the 6M and 3M groups, respectively).

The baseline demographic and disease characteristics of patients are summarized in Table 1. No major differences were observed in the baseline characteristics between the treatment groups.

**Table 1:** Baseline demographic and disease characteristics of patients (FAS)

Variable	Treatment group		Total (*n* = 167) *n* (%)
	TAP-144-SR (6M) (*n* = 83) *n* (%)	TAP-144-SR (3M) (*n* = 84) *n* (%)	
Age (years)			
≤39	13 (15.7)	12 (14.3)	25 (15.0)
40–44	29 (34.9)	30 (35.7)	59 (35.3)
≥45	41 (49.4)	42 (50.0)	83 (49.7)
Mean ± SD	44.2 ± 4.9	44.0 ± 5.2	44.1 ± 5.0
BMI (kg/m^2^)			
Mean ± SD	21.5 ± 3.0	21.5 ± 2.9	21.5 ± 3.0
Tumor stage (TNM classification)			
I	61 (73.5)	61 (72.6)	122 (73.1)
IIA	19 (22.9)	21 (25.0)	40 (24.0)
IIB	2 (2.4)	2 (2.4)	4 (2.4)
IIIA	1 (1.2)	0 (0.0)	1 (0.6)
Tumor size (cm)			
≤2.0	65 (78.3)	66 (78.6)	131 (78.4)
>2.0	18 (21.7)	18 (21.4)	36 (21.6)
Number of positive axillary lymph nodes			
0	68 (81.9)	70 (83.3)	138 (82.6)
1–3	15 (18.1)	14 (16.7)	29 (17.4)
ER/PgR expression			
ER+/PgR+	82 (98.8)	82 (97.6)	164 (98.2)
ER+/PgR−	1 (1.2)	2 (2.4)	3 (1.8)
ER−/PgR+	0 (0.0)	0 (0.0)	0 (0.0)
Radiation therapy			
Presence	52 (62.7)	59 (70.2)	111 (66.5)
Absence	31 (37.3)	25 (29.8)	56 (33.5)
Pre- and postoperative chemotherapy			
Presence	0 (0.0)	1 (1.2)	1 (0.6)
Absence	83 (100.0)	83 (98.8)	166 (99.4)
Serum estradiol (pg/mL) at Week 0			
Mean ± SD	168.0 ± 163.0	138.2 ± 125.5	153.0 ± 145.7

*FAS* full analysis set, *BMI* body mass index, *SD* standard deviation, *ER* estrogen receptor, *PgR* progesterone receptor

### Efficacy

#### E_2_ suppression rate

For the primary endpoint, the suppression rate of serum E_2_ to the menopausal level (≤30 pg/mL) from Week 4 through Week 48 in the FAS was 97.6 % (95 % CI 91.6–99.7) in the 6M group and 96.4 % (95 % CI 89.9–99.3) in the 3M group (Table 2). The estimated between-group difference in the suppression rate was 1.2 % (95 % CI −5.2 to 7.8). Since the lower CI was more than the pre-determined non-inferiority margin of −10 %, the non-inferiority of TAP-144-SR (6M) to TAP-144-SR (3M) was confirmed for the suppressive effect on serum E_2_. Five patients (2 and 3 patients in the 6M and 3M groups, respectively) had a serum E_2_ concentration exceeding 30 pg/mL during the period from the start of study drug administration to Week 48, which was measured at only 1 assessment time point in each patient. For the sensitivity analysis, the same analysis as for the primary analysis was utilized in the HAS as the secondary analysis. Similar results were obtained in the HAS [between-group difference in the suppression rate, 2.4 % (95 % CI −3.8 to 9.2)]. Therefore, the non-inferiority of TAP-144-SR (6M) to TAP-144-SR (3M) was confirmed in both analysis sets.

**Table 2:** Suppression rate of serum estradiol to the menopausal levels (≤30 pg/mL) from Week 4 through Week 48 (FAS)

	TAP-144-SR (6M) (*n* = 83)	TAP-144-SR (3M) (*n* = 84)
Suppression rate of serum estradiol [% (95 % CI)]	97.6 (91.6, 99.7)	96.4 (89.9, 99.3)
TAP-144-SR (6M) − TAP-144-SR (3M) [% (95 % CI)]	1.2 (−5.2, 7.8)	

*FAS* full analysis set, *E*_*2*_ estradiol, *CI* confidence interval

#### Changes in the hormone levels and menstrual status

**Fig. 2:**
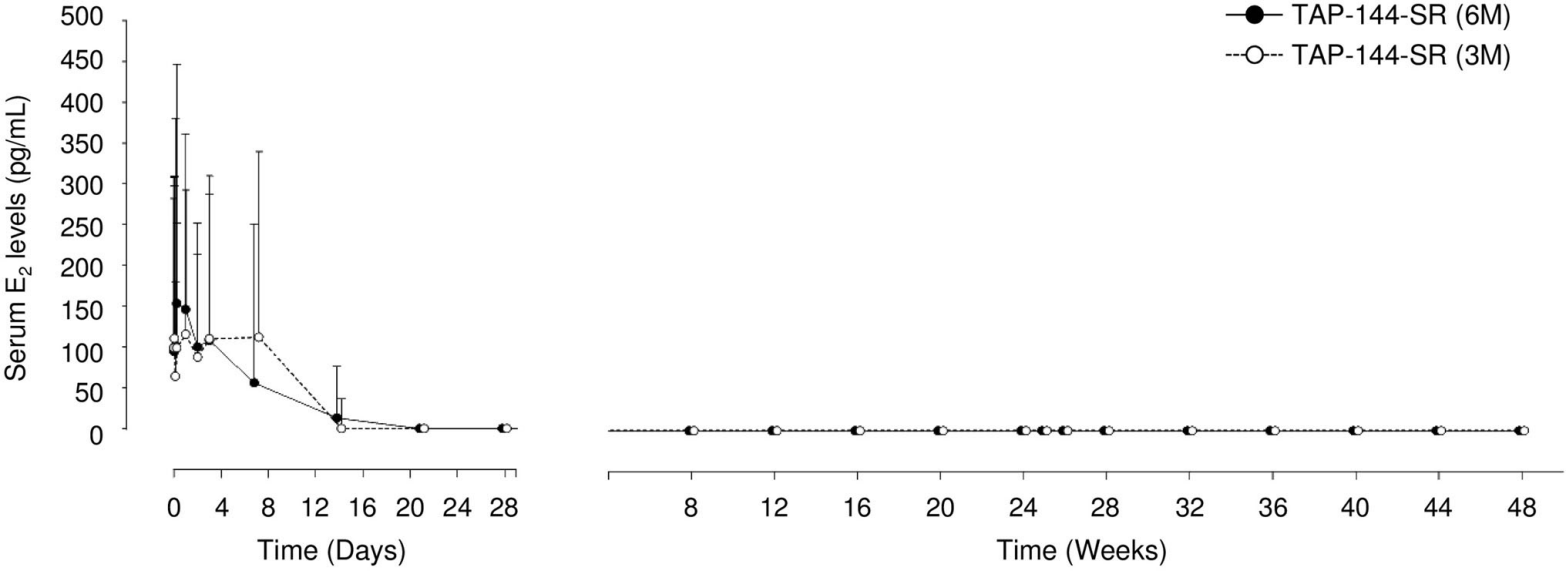
Time course of serum estradiol concentration from the start of study drug administration through Week 48 (FAS). Data are presented as the median and the 75th percentile. *E*_*2*_ estradiol, *FAS* full set analysis

The median serum E_2_ concentrations significantly declined to the value of 0 pg/mL, below the menopausal level of ≤30 pg/mL from Week 4 through Week 48 (Fig. 2), and remained at the suppressed level until Week 96 in both treatment groups.

Similarly, the median serum LH and FSH concentrations were suppressed to the levels of ≤1 and ≤2.5 mIU/mL, respectively from Week 4, and remained at the low levels through Week 96 in both treatment groups. There were no significant differences in the changes in these hormone levels between the treatment groups.

Throughout the study period, all patients achieved amenorrhea from Week 8, except 1 patient in the 6M group who had menses at Week 8.

#### DFS and DDFS

Throughout the 96-week study period, there were 4 disease events (2 each in the 6M and 3M groups, respectively): 3 recurrences (2 and 1), and 1 s primary cancer in the 3M group. One recurrence in the 6M group was bone metastasis. The DFS rate at Week 96 in the FAS was 97.3 % (95 % CI 93.6–100.0) and 97.5 % (95 % CI 94.1–100.0) in the 6M and 3M groups, respectively, with no significant between-group differences (estimated difference, −0.2 % [95 % CI −5.2 to 4.9]). The DDFS rate at Week 96 in the FAS was 98.5 % (95 % CI 95.7–100.0) and 98.8 % (95 % CI 96.4–100.0) in the 6M and 3M groups, respectively. There were no significant differences between the treatment groups (estimated difference, −0.3 % [95 % CI −4.0 to 3.4]).

### Pharmacokinetics

**Fig. 3:**
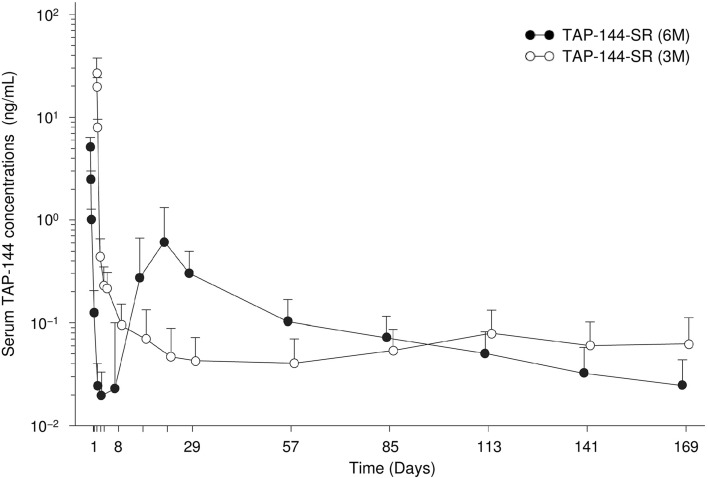
Time course of serum TAP-144 concentration from the start of study drug administration through week 24 (PAS). Data indicate the mean + SD. *SD* standard deviation, *PAS* pharmacokinetics analysis set

Serum TAP-144 concentrations rapidly increased immediately after the administration of TAP-144-SR (6M), and then rapidly decreased through Day 8 (Fig. 3). Thereafter, they increased again from Week 2 through Week 3, and gradually declined through Week 24, showing a double-peak of TAP-144. The profile of serum TAP-144 concentrations after the initial administration was similar to that after the second administration. In contrast, serum TAP-144 concentration rapidly increased 1 h after the administration of TAP-144-SR (3M), and then gradually declined during the period from 3 to 12 h. The maximum drug concentration (*C*_max_) in the 6M group was approximately one-fifth of that in the 3M group, and the area under the blood concentration–time curve in the 6M group was approximately 1.8 times that in the 3M group (data not shown). No obvious accumulation was observed either with TAP-144-SR (6M) or TAP-144-SR (3M).

### Safety

Throughout the study period, 98.8 % (82/83) and 97.6 % (82/84) of patients experienced AEs in the 6M and 3M groups, respectively The most common AEs were hot flush, followed by nasopharyngitis, radiation skin injury, injection site induration, injection site pain, white blood cell count decreased, headache and arthralgia, with no significant differences between the 2 groups (Table 3). The incidence of a series of injection site reactions (induration, pain, erythema, etc.) was 57.8 % (48/83) and 60.7 % (51/84) of patients in the 6M and 3M groups, respectively.

**Table 3:** Adverse events occurring in 10 % or more of patients in any treatment group (SAS)

Preferred term^a^	TAP-144-SR (6M) (*n* = 83) *n* (%)	TAP-144-SR (3M) (*n* = 84) *n* (%)
Patients with any AEs	82 (98.8)	82 (97.6)
Hot flush	43 (51.8)	48 (57.1)
Nasopharyngitis	47 (56.6)	42 (50.0)
Radiation skin injury	31 (37.3)	39 (46.4)
Injection site induration	36 (43.4)	33 (39.3)
Injection site pain	24 (28.9)	26 (31.0)
White blood cell count decreased	27 (32.5)	19 (22.6)
Headache	21 (25.3)	19 (22.6)
Arthralgia	18 (21.7)	20 (23.8)
Malaise	13 (15.7)	13 (15.5)
Injection site erythema	13 (15.7)	8 (9.5)
Musculoskeletal stiffness	11 (13.3)	9 (10.7)
Weight increased	12 (14.5)	8 (9.5)
Back pain	13 (15.7)	6 (7.1)
Insomnia	10 (12.0)	9 (10.7)
Injection site swelling	12 (14.5)	5 (6.0)
Hyperhidrosis	9 (10.8)	7 (8.3)
Nausea	7 (8.4)	9 (10.7)
Constipation	13 (15.7)	2 (2.4)
Dizziness	6 (7.2)	9 (10.7)
Gamma-glutamyltransferase increased	3 (3.6)	12 (14.3)
Rash	9 (10.8)	6 (7.1)
Eczema	3 (3.6)	9 (10.7)

*SAS* safety data analysis set, *AE* adverse event

^a^MedDRA, version 16.1

Most AEs were Grade 1 or 2 in severity. AEs of Grade 3 were reported in 14 (16.9 %) and 18 patients (21.4 %) in the 6M and 3M groups, respectively; AEs of Grade 4 were reported in 1 patient (1.2 %) in the 6M group. Drug-related AEs of ≥Grade 3 were anal fistula, blood triglycerides increased, liver function tests abnormal, hyperlipidaemia and interstitial lung disease (1 patient each) in the 6M group, and gamma-glutamyltransferase increased (3 patients), hypertension (2 patients), weight increased, neutropenia, blood triglycerides increased and interstitial lung disease (1 patient each) in the 3M group.

Serious AEs (SAEs) were reported in 6 (7.2 %) and 7 patients (8.3 %) in the 6M and 3M groups, respectively, and included 3 drug-related SAEs: interstitial lung disease in 1 patient in each group and anal fistula in 1 patient in the 6M group.

AEs leading to discontinuation of the study drug occurred in 4 (4.8 %) and 5 patients (6.0 %) in the 6M and 3M groups, respectively. Of these, AEs for which a causal relationship could not be ruled out were found in 4 patients (palpitations in 1 patient, joint stiffness, menopausal symptoms, and dry skin in 1, liver function test abnormal in 1, and interstitial lung disease in 1) in the 6M group, and in 4 patients (radiation pneumonitis in 1 patient, gamma-glutamyltransferase increased in 1, genital hemorrhage in 1, and interstitial lung disease in 1) in the 3M group. There were no deaths throughout the study period.

For ECG data analysis, QTcF intervals declined from the baseline values through 6 h after the study drug administration in both treatment groups, and no transient prolongation of QTcF intervals was detected at around the time of the *C*_max_. The mean changes (SD) in QTcF intervals from baseline were 9.7 (15.36) ms at Week 4, 9.2 (14.76) ms at Week 48, and 8.4 (14.73) ms at Week 96 in the 6M group, and 11.4 (13.83) ms at Week 4, 10.8 (15.13) ms at Week 48, and 13.1 (22.03) ms at Week 96 in the 3M group. Prolonged QTcF intervals of approximately 10 ms were observed from Week 4 through Week 96 in both treatment groups. Prolonged QTcF intervals of >60 ms from baseline were reported in 3 patients (2 and 1 in the 6M and 3M groups, respectively), which were transient and detected only at 1 assessment time point in each patient. At 7 institutions where it was possible to measure ECG at all assessment points, including 3 and 6 h after the study drug administration, the same ECG for all patients was used, and a total of 54 patients were interpreted at the central reading center. Prolonged QTcF intervals of approximately 15 ms were observed from Week 4 through Week 96 in both treatment groups. Similar prolonged QTcF intervals to those observed in the ECG measurements at the institutions were also observed in the measurements at the central reading center.

**Fig. 4:**
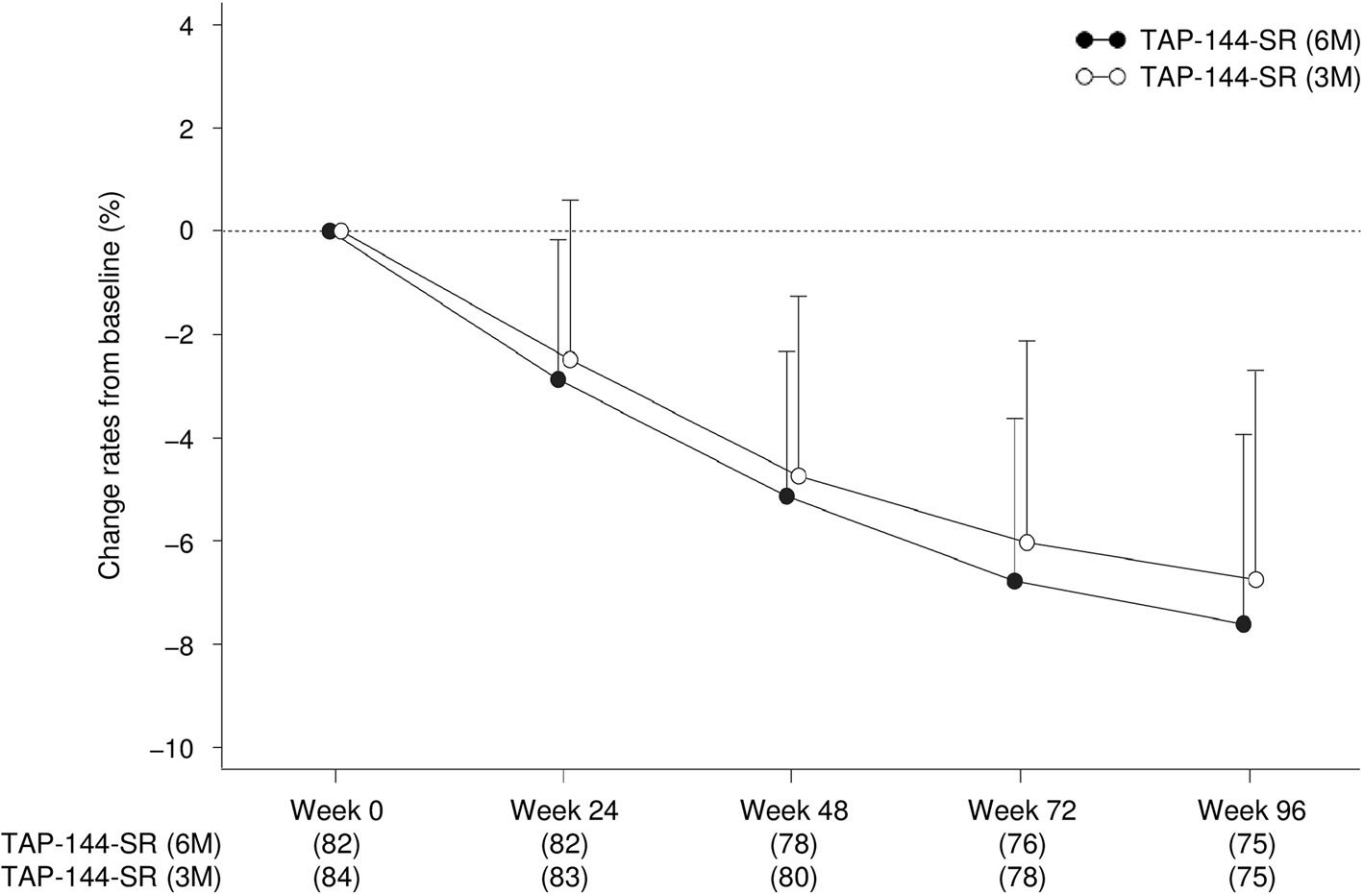
Time course of the mean change rates from baseline in bone mineral density of the lumbar spine in patients throughout the 96 week study period (SAS). Data indicate the mean + SD. *SD* standard deviation, *SAS* safety analysis set

For BMD of the lumbar spine (L_2_–L_4_), the mean change from baseline tended to gradually decline over time in both treatment groups. The mean change rate in BMD from baseline at Week 48 and Week 96 were −5.1 % (95 % CI −5.8 to −4.5) and −7.6 % (95 % CI −8.5 to −6.8) in the 6M group, and −4.7 % (95 % CI −5.5 to −4.0) and −6.7 % (95 % CI −7.7 to −5.8) in the 3M group. There were no significant differences in the BMD reduction between the treatment groups (Fig. 4).

## Discussion

This is the first report evaluating the efficacy and safety of 6-monthly injections of TAP-144-SR (6M) 22.5 mg in postoperative, premenopausal patients with hormone-receptor positive breast cancer. As for the primary endpoint, TAP-144-SR (6M) was non-inferior to TAP-144-SR (3M) for the effect to suppress the serum E_2_ to the menopausal level from Week 4 through Week 48.

TAP-144-SR (1M) and TAP-144-SR (3M) were effective to suppress the serum E_2_ level to a menopausal level in premenopausal breast cancer patients at the same dose at which suppression of serum testosterone to the castrate level was achieved in prostate cancer patients in clinical studies [14–16]. Therefore, the phase III study was conducted at the injection dose of TAP-144-SR (6M) 22.5 mg, which was determined in the phase II study for Japanese treatment-naïve prostate cancer patients [12]. The results showed that the injection dose of TAP-144-SR (6M) 22.5 mg, which was successfully used to suppress serum testosterone to the castrate level in prostate cancer patients [17], was also effective in premenopausal breast cancer patients (Fig. 2). In addition, TAP-144-SR (6M) was as effective as TAP-144-SR (3M) to suppress the serum LH and FSH levels from Week 4 through Week 96 in these patients. All patients also achieved amenorrhea from Week 8, except 1 patient in the 6M group who experienced menses at Week 8. It was therefore suggested that ovarian function was substantially suppressed by treatment with TAP-144-SR (6M) 22.5 mg every 24 weeks.

In this study, there were no significant differences in the DFS and DDFS at Week 96 between the groups. There are several reports that more than 3 or 4 years after surgery, the risk of recurrence is higher in ER-positive patients than ER-negative patients [18, 19], which may indicate that the risk of recurrence must be reduced by postoperative adjuvant hormone or chemotherapy for a longer period after surgery in ER-positive patients. At the St Gallen International Expert Consensus meetings, both postoperative tamoxifen and tamoxifen plus ovarian function suppression for 5 years were considered acceptable as the standard treatment for premenopausal patients with hormone-receptor positive breast cancer [10]. Furthermore, the results of the Suppression of Ovarian Function Trial (SOFT) showed that 5-year treatment with tamoxifen plus ovarian suppression might provide a benefit for DFS in high-risk patients younger than 35 years who have remained premenopausal after adjuvant chemotherapy, compared to tamoxifen alone [20]. Therefore, long-term administration of an LH–RH agonist is a possibility in high risk premenopausal patients with endocrine-responsive breast cancer.

In the PK analysis, TAP-144-SR (6M) showed a double-peak of TAP-144, similar to the results in the PK analysis obtained in previous Japanese clinical trials in prostate cancer patients [12], which demonstrated that the clinically sufficient drug concentration to suppress ovarian function was maintained throughout a period of 24 weeks with a single injection (Fig. 3). The serum TAP-144 concentration profile after the initial administration of TAP-144-SR (6M) was similar to that after its second administration.

As for safety, there were no significant differences in the safety profiles and tolerability between the groups, regarding the incidence, type and severity of AEs. The most common drug-related AEs included menopausal symptoms such as hot flush, headache and arthralgia, and injection site reactions (Table 3). A series of injection site reactions were reported in about 60 % of patients in each group with no significant between-group differences in the incidence and severity. All of these events were ≤Grade 2 in severity, and no cases led to discontinuation due to injection site reactions.

During the study period, 3 serious drug-related AEs were reported: interstitial lung disease in 1 patient in each treatment group and anal fistula in 1 patient in the 6M group. Both patients with interstitial lung disease had postoperative radiation and adjuvant endocrine therapy in the same period. It is known that pulmonary fibrosis frequently occurs following radiation therapy, and that tamoxifen may cause the development of lung fibrosis by inducing transforming growth factor-*β*. It was also reported that tamoxifen treatment during post-mastectomy radiation in breast cancer patients significantly increased the risk for the development of lung fibrosis along with other prognostic factors like age and menopausal status [21]. Since the pathogenesis of interstitial lung disease is still unclear, further research is necessary to evaluate whether the concomitant implementation of radiation and adjuvant TAP-144 plus tamoxifen therapy is associated with interstitial lung disease.

As for the ECG data, there was not a tendency of QTcF interval prolongation around the time of *C*_max_, and the mean change in QTcF intervals from Week 4 through Week 96 was approximately 10 ms in each treatment group. It is considered that this QTcF prolongation may not be primarily due to the pharmacological effect of TAP-144, but be secondary to the suppression of ovarian E_2_ production. A study of the effects of sex hormones on QTcF interval prolongation suggests that estrogen might be a risk factor for drug-induced torsades de pointes. Although E_2_ may influence clinically relevant QT interval prolongation, the pathogenesis is still not clear [22].

Although BMD tended to gradually decline over time in both treatment groups, the changes in BMD in TAP-144-SR (6M) were similar to those of the results of our previous clinical study in TAP-144-SR (3M) (Fig. 4) [23]. It is well recognized that serum E_2_ level suppression with an LH–RH agonist can cause BMD reduction, which can be prevented or mitigated with the concomitant use of anti-osteoporosis drugs [24].

In this phase III study, the non-inferiority of TAP-144-SR (6M) 22.5 mg to TAP-144-SR (3M) at 11.25 mg was confirmed in terms of the suppressive effect on serum E_2_ to the menopausal level. No clinically significant other differences in efficacy or tolerability were observed between the treatment groups.

TAP-144-SR (6M) allows mitigation of the burden on patients and physicians by reducing the dose frequency of the treatment for premenopausal patients with endocrine-responsive breast cancer. In particular, young premenopausal patients who are busy with work and housework may derive great benefit from TAP-144-SR (6M).
